# Autosomal recessive polycystic kidney disease: case report of a newborn with rare *PKHD1* mutation, rapid renal enlargement and early fatal outcome

**DOI:** 10.1186/s13052-020-00922-4

**Published:** 2020-10-15

**Authors:** Gregorio Serra, Giovanni Corsello, Vincenzo Antona, Maria Michela D’Alessandro, Nicola Cassata, Marcello Cimador, Mario Giuffrè, Ingrid Anne Mandy Schierz, Ettore Piro

**Affiliations:** 1 Department of Health Promotion, Mother and Child Care, Internal Medicine and Medical Specialties “G. D’Alessandro”, University Hospital “P.Giaccone”, Palermo, Italy; 2 Department of Pediatric Nephrology, Children’s Hospital “G. Di Cristina”, Palermo, Italy; 3 Department of Pediatrics, A.O. Ospedali Riuniti Villa Sofia-Cervello, Palermo, Italy

**Keywords:** ARPKD, Potter sequence, Next generation sequencing, Genotype-phenotype correlation, Ethics

## Abstract

**Introduction:**

Autosomal recessive polycystic kidney disease (ARPKD; MIM#263200) is one of the most frequent pediatric renal cystic diseases, with an incidence of 1:20,000. It is caused by mutations of the *PKHD1* gene, on chromosome 6p12. The clinical spectrum is highly variable, ranging from late-onset milder forms to severe perinatal manifestations. The management of newborns with severe pulmonary insufficiency is challenging, and causes of early death are sepsis or respiratory failure. In cases of massive renal enlargement, early bilateral nephrectomy and peritoneal dialysis may reduce infant mortality. However, there is no conclusive data on the role of surgery, and decision-making is driven by patient’s clinical condition and expertise of the center.

**Patient presentation:**

We hereby describe a preterm female newborn with perinatal, rapid and bilateral, abnormal growth of both kidneys, respiratory failure and initial signs of liver disease. She was subsequently confirmed to be affected by a rare and severe homozygous mutation of the *PKHD1* gene, inherited from both her consanguineous parents. Our patient died 78 days after birth, due to a fungal sepsis which worsened her respiratory insufficiency.

**Conclusions:**

This patient report shows some of the clinical and ethical issues of neonatal ARPKD, and the need of multidisciplinary approach and good communication with the family. Target next generation sequencing (NGS) techniques may guide and support clinicians, as well as guarantee to these patients the most appropriate clinical management, avoiding unnecessary and/or disproportionate treatments.

## Introduction

Autosomal recessive polycystic kidney disease (ARPKD; MIM#263200) is one of the most frequent pediatric renal cystic diseases, with an incidence of 1:20,000 [1]. It is caused by mutations of the *PKHD1* gene (polycystic kidney and hepatic disease-1, also known as ciliary IPT domain containing fibrocystin/polyductin) on chromosome 6p12. The clinical spectrum is highly variable, ranging from late-onset milder forms, to severe perinatal manifestations (more often associated with truncating *PKHD1* changes) [2]. The management of newborns with severe pulmonary insufficiency is particularly challenging, also due to lack of reliable clinical prognostic markers [3, 4]. Causes of early death are sepsis or respiratory failure, due to lung hypoplasia and displacement of the diaphragm by bilateral nephromegaly [2]. In cases of massive renal enlargement, early bilateral nephrectomy, supportive peritoneal dialysis and early aggressive nutrition may reduce infant mortality [3]. However, there is no conclusive data on role and optimal timing of surgery, and decision-making is driven by patient’s clinical condition and expertise of the center [3]. We hereby describe a female preterm newborn with perinatal, rapid and bilateral, abnormal growth of both kidneys, respiratory failure and initial signs of liver disease. She was subsequently confirmed to be affected by a rare, and severely truncating, homozygous mutation of the *PKHD1* gene, inherited from both her consanguineous parents.

## Patient presentation

**Fig. 1:**
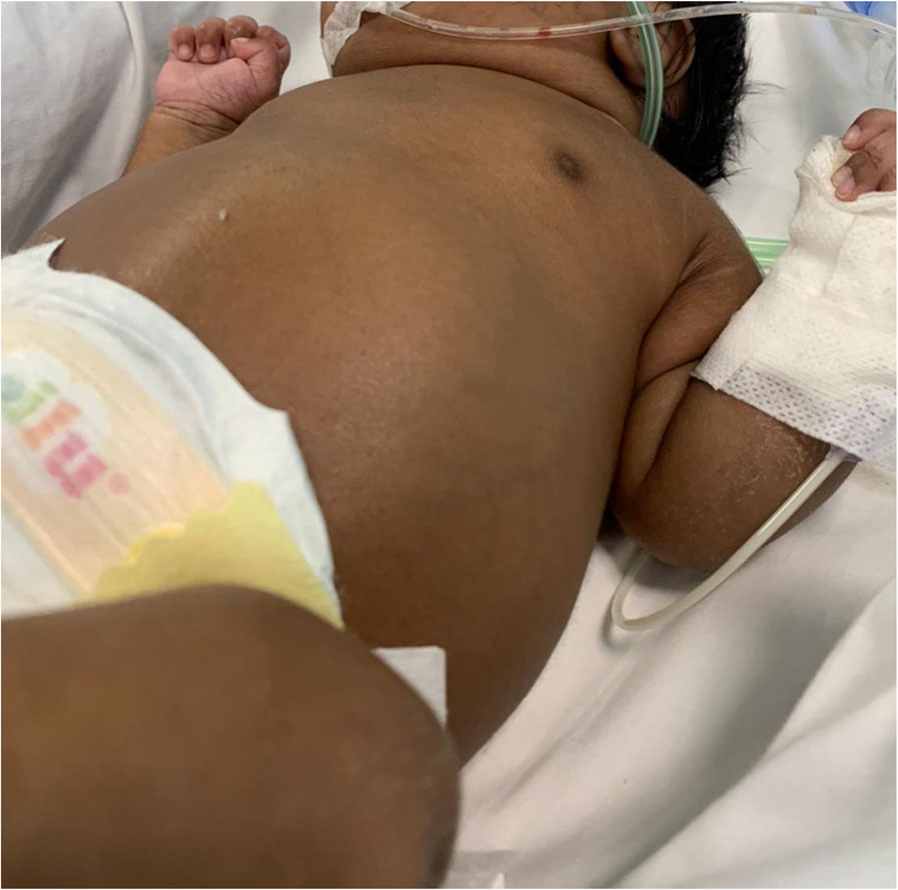
Partial profile of the patient, severe abdominal distension with bilateral palpable nephromegaly

A female newborn was delivered at 33^+ 4^ weeks of gestation, by cesarean section for preterm labour and oligohydramnios. Her parents were first-grade cousins originating from Bangladesh. Both, and the two-year-old sister, showed normal renal and hepatic ultrasonography (US) and function tests. Family history was uneventful, while pregnancy was remarkable for pregestational diabetes, requiring insulin treatment. Prenatally, oligohydramnios without any enlargement and/or increased echogenicity of kidneys was observed. At birth, anthropometric measurements were as follows: weight 2170 g (98th centile), length 46 cm (84th centile), and occipitofrontal circumference 31 cm (58th centile). Apgar scores were 8 and 9 at 1 and 5 min, respectively. On physical examination she showed the typical Potter sequence face (flattened nose, micrognathia, large and low-set ears), and severe abdominal distension with bilateral palpable nephromegaly (Fig. 1). Redundant skin of the neck, axial hypotonia and limb contractures were also observed.

**Fig. 2:**
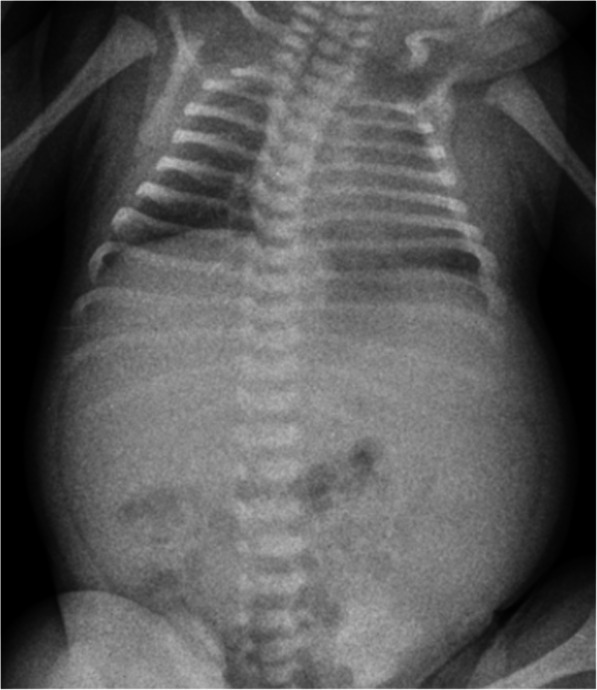
X-ray: lung hypoplasia, with reduced expansion and diffuse bilateral accentuated bronchovascular markings, and severe abdominal distension due to visceromegaly (liver and kidneys)

**Fig. 3:**
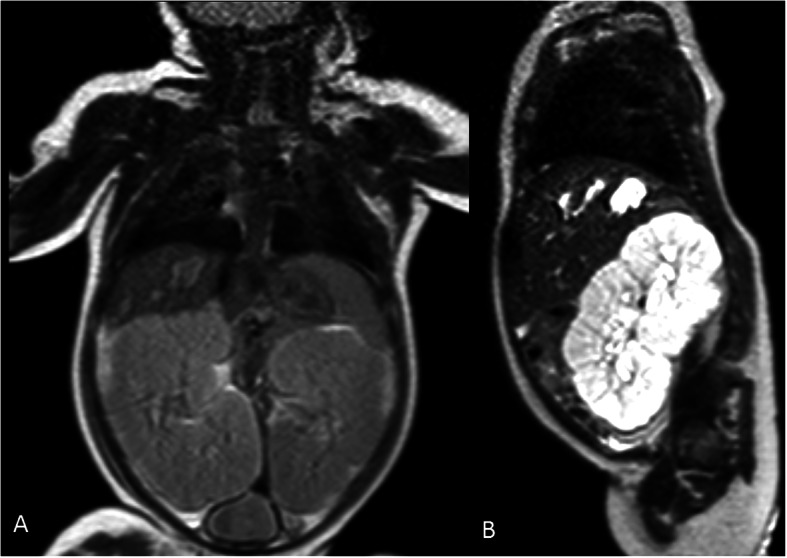
Abdominal magnetic resonance imaging. **a**. T1 sequence, in coronal plane, shows enlargement of both kidneys (right 8.5 × 5.4 cm, left 8.2 × 5.4 cm), with altered structure and bulged margins, and hepatomegaly with at least two cystic lesions in segments VII and VIII. **b**. T2 sequence delayed enhanced image, in right parasagittal plane, shows enlarged kidney with inverted structure, and at least two cystic focal lesions in segments VII and VIII of the liver

Postnatally, she suffered from severe respiratory distress syndrome due to pulmonary hypoplasia, which needed intubation, surfactant administration and invasive mechanical ventilation (MV, peak inspiratory pressure up to 26 cmH_2_O) for 10 days. Chest X-ray confirmed the typical patterns of lung hypoplasia (Fig. 2). On day 1, abdominal US revealed bilateral cystic nephromegaly, with the right kidney measuring 5.5 cm in length, and the left 6.2 cm. Renal function was moderately impaired, with glomerular filtration rate, obtained by the Schwartz formula, of 10.12 mL/min/1.73m^2^ (k = 0.33). Oliguria (urine output below 1 ml/kg/h) was present in the first hours of life, and required, soon after birth, a progressively increasing dosage of diuretics and dopamine. Moreover, the newborn showed increased urinary excretion of sodium, and decreased of potassium, with consequent hyponatremia and hyperkalemia, that were corrected with sodium chloride and a fluid restriction regimen. A rapid and progressive enlargement of both kidneys (maximum longitudinal diameter of around 8.5 cm for both) was noted on US, till the third week of life. Renal morphology was characterized by bilateral poor corticomedullary differentiation, and typical pepper-salt pattern (diffusely increased parenchymal echogenicity, with small medullary cysts and fusiform dilatation of collecting ducts) [5]. At this time, around 20 days of life, she developed severe hypertension, with maximum systolic blood pressure 137 mmHg and mean arterial pressure 129 mmHg (> 95th centile). However, antihypertensive treatment with intravenous amlodipine and furosemide with progressively increasing doses (maximum 0.4 mg/kg/day and 4 mg/kg/day, respectively), was not effective to achieve blood pressure control. Magnetic resonance imaging of the abdomen, performed at 2 months of age, confirmed the enlargement of both kidneys (right 8.5 × 5.4 cm, left 8.2 × 5.4 cm), with altered structure and bulged margins, and decreased kidney function (mild and inhomogeneous medullary impregnation of the contrast medium, without evident cortical and excretory phase), which was further showed by renal radionuclide imaging. An increased size of the liver was also observed, as well as peripheral biliary dilatation in the right lobe and at least two cystic lesions in segments VII and VIII (Fig. 3).

Establishing enteral feeding was particularly challenging during the whole hospital stay, due to the abdominal mass compression. However, a nasojejunal feeding tube was inserted, although it was maintained for only 4 days. Then, owing to the failure to ensure an effective tube feeding regimen, a total parenteral nutrition (TPN) was started.

The newborn did not need any ventilator support until the 70th day of life, when, as a consequence of renal enlargement, anemia and increasing edema, due to both hypoproteinemia and severe hypothyroidism, MV became again necessary. Echocardiogram, at this point, evidenced left ventricular and interventricular septal hypertrophy, as well as dilatation of the arterial coronaries and pulmonary hypertension (PAPs 35 mmHg).

As the patient remained MV and TPN dependent, it was discussed the option to proceed with nephrectomy. The parents were fully counseled regarding the benefits/risks ratio and prognosis as well as the available options like surgical intervention, dialysis, including issues relating to transplantation and potential lifetime dependency [6], or palliative care. Multiprofessional and multidisciplinary discussions including bioethical aspects, also with the support of a cultural mediator, were then made, taking into account parents’ will. At the end of the consultation, the parents agreed with active management.

However, a quick decay of the clinical conditions occurred. Indeed, our patient died on the 78th day of life, with a fungal sepsis which definitely worsened her respiratory insufficiency.

Meanwhile, the genomic diagnosis of ARPKD by target NGS was obtained, and confirmed after Sanger sequencing. The homozygous mutation NM_138694.4:c.5323C > T (p.Arg1775Ter) of the *PKHD1* gene was identified in the proband. Such variant, rarely associated with ARPKD [7–9], was inherited by both parents, who are heterozygous carriers of the same mutation. It causes a premature stop codon, at the amino acid 1775 of the encoded protein (which normally contains 4074 amino acids), leading thus to a severe truncation.

## Discussion and conclusions

In 1994, the causative gene for ARPKD, *PKHD1*, was mapped to chromosome 6p12. However, only in recent years it was sequenced, allowing, thus, to achieve the genomic profile of the affected patients, as well as the differential diagnosis from other renal cystic diseases [10–13].

The majority of ARPKD patients are identified late in pregnancy or at birth. Even second trimester fetal US often fails to detect enlargement and/or increased echogenicity of both kidneys [10, 14], and oligohydramnios may be the only sign of the disease, as occurred in the present patient. Newborns with perinatally diagnosed massive renal enlargement have a poor prognosis, and usually die of respiratory problems within the first days of life [2, 15, 16].

The exceptionally rapid enlargement of the kidneys described in the present report, raises relevant questions about the pathogenic mechanisms underlying the disease. Specifically, the rare homozygous mutation of our proband showed to be particularly severe, as it is a nonsense variant, which inserts a stop codon within the amino acid sequence of the encoded protein (polyductin/fibrocystin, 4074 amino acids). Indeed, the *PKHD1* transcript is interrupted at the amino acid 1775, and lacks its major part. This suggests a severe functional impairment of the protein, and may explain the clinical severity and the unfavorable outcome of the *proposita*. However, the serious clinical evolution of our newborn may also be related to other factors added to the *PKHD1* genotype (as supported by the reported intrafamilial clinical variability among affected siblings) [2]. It may also depend on other genetic and/or epigenetic factors [17]. Thus, the severity of the disease is attributable to both modifier genes, and environmental factors. In our patient glycometabolic derangements, related to maternal diabetes, could have exerted deleterious effects both before conception, altering the biologic/molecular features of maternal germinal cells, and during embryo-fetal development [2, 18, 19].

From a biochemical/molecular point of view, it has been supposed that proteins, like the epidermal growth factor receptor (EGFR), are abnormally expressed in the cystic renal epithelia of such patients. Their increased expression is associated with epithelial hyperplasia, leading to progressive enlargement of cysts [2]. Moreover, in these subjects it has been observed an increased activation of the mTOR (mechanistic target of rapamycin kinase) signaling pathway [20]. Both mechanisms, shared with other diseases, might explain the extremely high level of proliferation of the cystic epithelia, which led to the severe phenotype in our patient as well as in those with overlapping clinical features. Also, pulmonary hypertension, reported in some ARPKD patients [2] and present in our newborn, may recognize such biochemical/molecular alterations. Thus, it may be associated, aside from the condition of chronic hypoxia, with endothelial dysfunction and hypercontraction of vascular smooth muscle cells [2].

Despite recent advances in neonatal care, genetic knowledge, and excellent outcomes for pediatric kidney transplant, the 1-year mortality is still to date around 30% [21], and more often related to pulmonary hypoplasia, rather than to renal insufficiency [3]. MV is a strong negative predictor of long-term survival, and patients who need ventilator support, like our newborn, show a higher mortality rate than those who develop hypertension, progressive renal insufficiency, and portal tract fibrosis [10].

Unilateral or bilateral nephrectomy, with subsequent peritoneal dialysis, have been reported as therapeutic approach for selected subjects [2].

The management of the severely affected neonates, therefore, focuses on MV and, occasionally, on unilateral or bilateral nephrectomy [3]. Indeed, early removal of both fast-growing kidneys, pre-emptive peritoneal dialysis, since haemodialysis may be considered only when the peritoneal one is impossible to perform [2], and kidney transplantation, may be the most promising option in patients with respiratory impairment due to increased peri/postnatal growth of the kidneys [10]. However, the risk of accelerating renal function loss, and the consequent need for renal replacement therapy early in life, must be carefully weighed [3, 22]. In our patient, the fulminant worsening of the clinical conditions did not allow for an effective treatment plan. Indeed, the present patient shows some of the clinical and bioethical issues, also related to the difficulties encountered due to cultural and linguistic differences, which may arise in the management of ARPKD newborns.

This patient report may provide further insights into the molecular pathogenesis of ARPKD, as well as a better genomic and clinical characterization of the disease. The study of new patients will allow to outline further genotype-phenotype correlations [23].

The hope for the next future may come from the ever-deeper knowledge of the underlying genomic/molecular mechanisms, which may lead to effective therapies [24] (i.e. the inhibition of mTOR to attenuate the growth of cystic epithelium, potentially useful also for other pathologies) [25, 26].

Meanwhile, the role played by next generation sequencing (NGS) techniques is becoming increasingly precious and determinant. These methods, providing precise genomic information, make the use of invasive investigations, like renal biopsy in ARPKD, no longer necessary, and allow to define more accurate prognoses. Indeed, when promptly obtained, they may guide and support clinicians and guarantee to the patients the most appropriate clinical management, avoiding futile and/or disproportionate treatments, as well as further unnecessary separations between children and their parents [27]. Then, in critical situations, as in our patient, the attention may shift from the invasive approach to a different goal, which is the reduction in suffering.

## Supplementary information

**Additional file 1.**

## Data Availability

The datasets used and analyzed during the current study are available from the corresponding author on reasonable request.

## Abbreviations

ARPKD: Autosomal recessive polycystic kidney disease
EGFR: Epidermal growth factor receptor
MV: Mechanical ventilation
mTOR: Mechanistic target of rapamycin kinase
NGS: Next generation sequencing
PAPs: Systolic pulmonary artery pressure
PKHD1: Polycystic kidney and hepatic disease-1
TPN: Total parenteral nutrition
US: Ultrasonography

## References

[1] Bergmann C (2019). Early and severe polycystic kidney disease and related Ciliopathies: an emerging field of interest. Nephron..

[2] Arbeiter A, Büscher R, Bonzel KE, Wingen AM, Vester U, Wohlschläger J, Zerres K, Nürnberger J, Bergmann C, Hoyer PF (2008). Nephrectomy in an autosomal recessive polycystic kidney disease (ARPKD) patient with rapid kidney enlargement and increased expression of EGFR. Nephrol Dial Transplant.

[3] Mallett TM, O'Hagan E, McKeever KG (2015). Early Bilateral Nephrectomy in Infantile Autosomal Recessive Polycystic Kidney Disease. BMJ Case Rep.

[4] Abbiss H, Maker GL, Trengove RD (2019). Metabolomics approaches for the diagnosis and understanding of kidney diseases. Metabolites..

[5] Iorio P, Heidet L, Rutten C, Garcelon N, Audrézet MP, Morinière V, Boddaert N, Salomon R, Berteloot L (2020). The "salt and pepper" pattern on renal ultrasound in a Group of Children with Molecular-Proven Diagnosis of Ciliopathy-related renal diseases. Pediatr Nephrol.

[6] Burgmaier K, Kunzmann K, Ariceta G, Bergmann C, Buescher AK, Burgmaier M, Dursun I, Duzova A, Eid L, Erger F, Feldkoetter M, Galiano M, Geßner M, Goebel H, Gokce I, Haffner D, Hooman N, Hoppe B, Jankauskiene A, Klaus G, König J, Litwin M, Massella L, Mekahli D, Melek E, Mir S, Pape L, Prikhodina L, Ranchin B, Schild R, Seeman T, Sever L, Shroff R, Soliman NA, Stabouli S, Stanczyk M, Tabel Y, Taranta-Janusz K, Testa S, Thumfart J, Topaloglu R, Weber LT, Wicher D, Wühl E, Wygoda S, Yilmaz A, Zachwieja K, Zagozdzon I, Zerres K, ESCAPE Study Group (2018). GPN Study Group; Dötsch J, Schaefer F, Liebau MC, ARegPKD consortium. Risk Factors for Early Dialysis Dependency in Autosomal Recessive Polycystic Kidney Disease. J Pediatr.

[7] Melchionda S, Palladino T, Castellana S, Giordano M, Benetti E, De Bonis P, Zelante L, Bisceglia L (2016). Expanding the mutation Spectrum in 130 Probands with ARPKD: identification of 62 novel PKHD1 mutations by sanger sequencing and MLPA analysis. J Hum Genet.

[8] Obeidova L, Seeman T, Elisakova V, Reiterova J, Puchmajerova A, Stekrova J (2015). Molecular genetic analysis of PKHD1 by next-generation sequencing in Czech families with autosomal recessive polycystic kidney disease. BMC Med Genet.

[9] Bergmann C, Senderek J, Windelen E, Küpper F, Middeldorf I, Schneider F, Dornia C, Rudnik-Schöneborn S, Konrad M, Schmitt CP, Seeman T, Neuhaus TJ, Vester U, Kirfel J, Büttner R, Zerres K (2005). APN (Arbeitsgemeinschaft für Pädiatrische Nephrologie). Clinical consequences of PKHD1 mutations in 164 patients with autosomal-recessive polycystic kidney disease (ARPKD). Kidney Int.

[10] Prelog M, Bergmann C, Ausserlechner MJ, Fischer H, Margreiter R, Gassner I, Brunner A, Jungraithmayr TC, Zerres K, Sergi C, Zimmerhackl LB (2006). Successful transplantation in a child with rapid progression of autosomal recessive polycystic kidney disease associated with a novel mutation. Pediatr Transplant.

[11] Bergmann C, Küpper F, Schmitt CP, Vester U, Neuhaus TJ, Senderek J, Zerres K (2005). Multi-exon deletions of the PKHD1 gene cause autosomal recessive polycystic kidney disease (ARPKD). J Med Genet.

[12] Bergmann C (2015). ARPKD and early manifestations of ADPKD: the original polycystic kidney disease and Phenocopies. Pediatr Nephrol.

[13] Bergmann C (2018). Genetics of autosomal recessive polycystic kidney disease and its differential diagnoses. Front Pediatr.

[14] Erger F, Ortiz-Brüchle N, Gembruch U, Zerres K (2017). Prenatal ultrasound, genotype, and outcome in a large cohort of prenatally affected patients with autosomal-recessive polycystic kidney disease and other hereditary cystic kidney diseases. Arch Gynecol Obstet.

[15] Luo F, Gu WZ, Chen Z, Shi LP, Ma XL, Lin HJ, Qiu YH (2013). Infantile polycystic kidney disease: a case report and literature review. Zhonghua Er Ke Za Zhi.

[16] Khare A, Krishnappa V, Kumar D, Raina R (2018). Neonatal renal cystic diseases. J Matern Fetal Neonatal Med.

[17] Corsello G, Salzano E, Vecchio D, Antona V, Grasso M, Malacarne M, Carella M, Palumbo P, Piro E, Giuffrè M (2015). Paternal Uniparental Disomy chromosome 14-like syndrome due a maternal De novo 160 kb deletion at the 14q32.2 region not encompassing the IG- and the MEG3-DMRs: patient report and genotype-phenotype correlation. Am J Med Genet A.

[18] Serra G, Antona V, Schierz M, Vecchio D, Piro E, Corsello G (2018). Esophageal atresia and Beckwith–Wiedemann syndrome in one of the naturally conceived discordant newborn twins: first report. Clin Case Rep.

[19] Serra G, Antona V, Corsello G, Zara F, Piro E, Falsaperla R (2019). NF1 microdeletion syndrome: case report of two new patients. Ital J Pediatr.

[20] Fischer DM, Jacoby U, Pape L, Ward CJ, Kuwertz-Broeking E, Renken C, Nizze H, Querfeld U, Rudolph B, Mueller-Wiefel DE, Bergmann C, Haffner D (2009). Activation of the AKT/mTOR pathway in autosomal recessive polycystic kidney disease (ARPKD). Nephrol Dial Transplant.

[21] Meliţ LE, Mărginean CO, Mărginean CD, Mărginean MO, Aldea C (2019). Neonatal polycystic kidney disease, a potential life-threatening condition at this age: a case report. Medicine (Baltimore).

[22] Riechardt S, Koch M, Oh J, Fisch M (2017). Early bilateral nephrectomy in neonatal autosomal recessive polycystic kidney disease: improved prognosis or unnecessary effort?. Urologe A.

[23] Hou L, Du Y, Zhang M, Su P, Zhao C, Wu Y (2018). Novel mutations of PKHD1 and AHI1 identified in two families with cystic renal disease. Int J Clin Exp Pathol.

[24] Chang MY, Ong ACM (2018). Targeting new cellular disease pathways in autosomal dominant polycystic kidney disease. Nephrol Dial Transplant.

[25] Corsello G, Antona V, Serra G, Zara F, Giambrone C, Lagalla L, Piccione M, Piro E (2018). Clinical and molecular characterization of 112 single-center patients with Neurofibromatosis type 1. Ital J Pediatr.

[26] Nowak KL, Edelstein CL (2020). Apoptosis and autophagy in polycystic kidney disease (PKD). Cell Signal.

[27] Obeidova L, Seeman T, Fencl F, Blahova K, Hojny J, Elisakova V, Reiterova J, Stekrova J (2020). Results of targeted next-generation sequencing in children with cystic kidney diseases often change the clinical diagnosis. PLoS One.

