# Supplementary material for: Autosomal recessive polycystic kidney disease: case report of a newborn with rare PKHD1 mutation, rapid renal enlargement and early fatal outcome
Source: Ital J Pediatr. 2020 Oct 15;46:154. doi: 10.1186/s13052-020-00922-4 (PMC7560064; doi:10.1186/s13052-020-00922-4)
Supplement: Supplementary file 1 — Additional file 1. [file 13052_2020_922_MOESM1_ESM.doc]

parents were first-grade cousins

parents (first grade cousins) and the two-year-old sister: normal renal and hepatic US and function tests

Pregestational diabetes, oligohydramnios

Preterm birth at 33+^4^ weeks

weight 2170 g (98^th^ centile)

typical Potter sequence face (flattened nose, micrognathia, large and low-set ears)

severe abdominal distension with bilateral palpable nephromegaly

redundant skin of the neck, axial hypotonia and limb contractures

Postnatally, severe respiratory distress syndrome: MV

In the first hours of life, impaired renal function and oliguria

20^th^ day

severe hypertension

20 days of life severe hypertension

60^th^ day

target NGS: homozygous c.5323C>T mutation of the *PKHD1* gene

78^th^ day

death for a fungal sepsis worsening her respiratory insufficiency

70^th^ day

Renal enlargement, anemia and increasing edema: MV again necessary
